# Surgical treatment of the anomalous origin of the right pulmonary artery from the ascending aorta: report of two cases

**DOI:** 10.47487/apcyccv.v6i4.549

**Published:** 2025-12-29

**Authors:** Silvia Lisseth Ocampo Quito, Gabriela Susana López Lavado, Edwin Martin Bedoya Rivera, Luis Alfredo Pacheco Ramos

**Affiliations:** 1 Instituto Nacional de Salud del Niño San Borja, Lima, Perú. Instituto Nacional de Salud del Niño San Borja Lima Perú

**Keywords:** Pulmonary Artery, Heart Defects, Congenital, Heart Failure, Echocardiography, Cardiac Surgical Procedures, Arteria Pulmonar, Cardiopatías Congénitas, Insuficiencia Cardíaca, Ecocardiografía, Procedimientos Quirúrgicos Cardíacos

## Abstract

The anomalous origin of the right pulmonary artery is a rare congenital cardiac malformation that leads to early development of pulmonary vascular disease, heart failure, and death. Therefore, surgical correction should be performed as soon as the diagnosis is established. A high index of clinical suspicion and the use of non-invasive imaging studies play a crucial role in early diagnosis and timely intervention, thereby reducing the high mortality rates associated with this congenital heart disease. We present two cases of this rare entity, both with clinical manifestations of heart failure from the neonatal period. In both cases, surgical correction was performed through direct anastomosis of the right pulmonary artery to the main pulmonary artery. Both patients showed a favorable postoperative course, with no clinical or echocardiographic evidence of anastomotic stenosis or pulmonary hypertension.

## RESUMEN

El origen anómalo de la arteria pulmonar derecha es una malformación cardiaca poco frecuente que condiciona el desarrollo temprano de enfermedad vascular pulmonar, falla cardiaca y muerte. Por lo tanto, la resolución quirúrgica debe realizarse una vez hecho el diagnóstico. Un alto índice de sospecha clínica y los estudios de imagen no invasivos cumplen una función importante en el diagnóstico precoz e intervención temprana, con la consecuente reducción de las altas tasas de mortalidad asociadas a esta cardiopatía. Presentamos dos casos de esta rara entidad, con presentación clínica de insuficiencia cardiaca desde la etapa neonatal; en ambos casos se realizó la corrección quirúrgica mediante anastomosis directa de la arteria pulmonar derecha al tronco de la pulmonar. Los pacientes presentaron una evolución posoperatoria favorable sin datos clínicos ni ecocardiográficos de estenosis en las zonas de anastomosis o hipertensión pulmonar.

## Intoduction

The anomalous origin of one of the pulmonary arteries is a rare congenital cardiac malformation, first described by Fraentzel O. in 1868 ^1^, in which either the right or left pulmonary artery arises from the ascending aorta ^2^. Its reported prevalence ranges from 0.05% to 0.33% ^3^^-^^5^, and the most common form is the anomalous origin of the right pulmonary artery from the ascending aorta, known as AORPA.

Although uncommon, this condition warrants careful documentation to improve understanding of its marked haemodynamic impact, as the pulmonary vascular bed is exposed to pressure and volume overload ^2^^,^^4^^,^^6^^,^^7^. Affected patients typically present with signs of congestive heart failure at an early age ^2^^,^^8^, and the natural history is characterised by the early development of pulmonary vascular disease and heart failure, with reported mortality rates of 70% at 6 months and 80% at 1 year of life ^4^^,^^6^^,^^9^. The presentation of these two cases provides an opportunity to analyse the clinical features, underscore the importance of early diagnosis through accurate echocardiographic interpretation, and highlight the complementary role of computed tomography or magnetic resonance imaging in better delineating the anatomical details ^5^. It also emphasises the importance of timely surgical intervention, for which several techniques have been described to reimplant the anomalous pulmonary artery into the pulmonary trunk, including direct implantation ^2^^,^^10^^,^^11^, the approach used in our patients. Outcomes following early intervention are generally favourable; however, overall in-hospital mortality ranging from 0% to 21% has been reported ^1^^,^^9^. Postoperative complications include bleeding, low cardiac output syndrome, pulmonary hypertension, atrioventricular block, and even death ^12^^-^^14^. In the long term, surgical or percutaneous reintervention may be required in 12.5% to 36% of cases to address supravalvular aortic stenosis or stenosis of the reimplanted pulmonary artery ^9^.

## Case report

### Case 01

**Figure 1:**
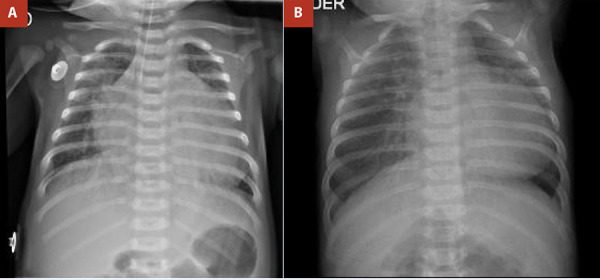
Chest radiograph. **A)** Case 01. **B)** Case 02. Dilatation of the left cardiac chambers with bilateral pulmonary overcirculation.

**Figure 2:**
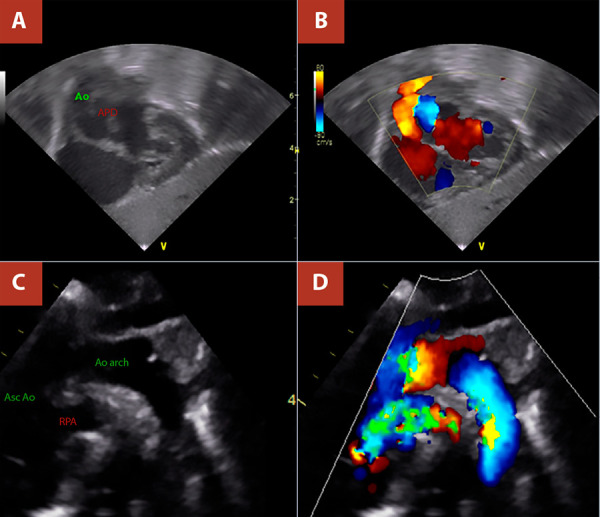
Transthoracic echocardiography, Case 1. **A)** and **B)** Subcostal views showing the origin of the right pulmonary artery from the ascending aorta. **C)** and **D)** Suprasternal views demonstrating the origin of the right pulmonary artery from the posterior wall of the ascending aorta. RPA: right pulmonary artery.

**Figure 3:**
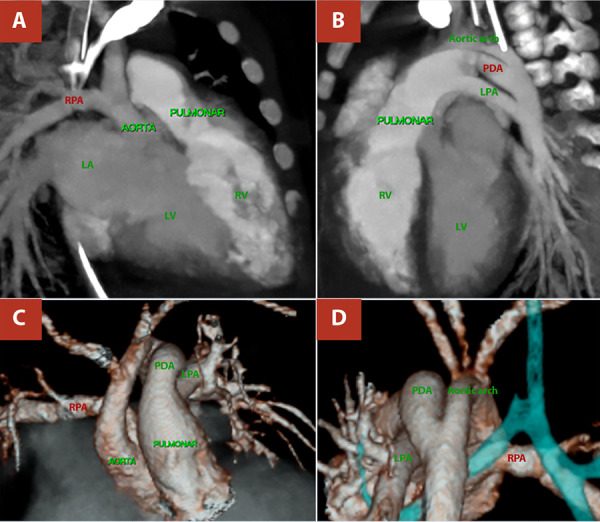
Cardiac and great-vessel computed tomographic angiography, Case 01. **A)** Anomalous origin of the right pulmonary artery (RPA) from the posterior wall of the ascending aorta. **B)** Origin of the left pulmonary artery (LPA) from the main pulmonary artery and presence of a patent ductus arteriosus (PDA). **C)** and **D)** Three-dimensional reconstruction of the heart and great vessels confirming the findings.

A term newborn female presented with respiratory distress from the fifth day of life, associated with cyanosis and interrupted breastfeeding, and was initially managed as neonatal sepsis. She was referred to our centre at 19 days of life because of suspected congenital heart disease. On admission, vital signs included a heart rate of 138 beats per minute, respiratory rate of 45 breaths per minute, and oxygen saturation of 80%. Physical examination revealed subcostal retractions and a cardiac murmur on auscultation. Chest radiography showed biventricular cardiomegaly with bilateral pulmonary overcirculation (Figure 1). Electrocardiography demonstrated sinus rhythm with a QRS axis of +120°. Transthoracic echocardiography revealed the emergence of a vessel with apparent bifurcation arising from the left ventricle and a continuous vessel originating from the right ventricle, raising initial suspicion of transposition of the great arteries (TGA); consequently, prostaglandin E1 (PGE1) infusion was initiated. On repeat echocardiographic assessment, with clearer identification of coronary artery origins and absence of the classic parallel vessel arrangement characteristic of TGA, the diagnosis was revised to AORPA (Figure 2). Additional findings included signs of pulmonary hypertension, a 5-mm atrial septal defect, and a 4-mm patent ductus arteriosus; these findings were corroborated by computed tomography (Figure 3). PGE1 was discontinued, and the patient underwent surgical correction at 37 days of life, after completion of treatment for sepsis.

During her stay in the intensive care unit (ICU), she developed low cardiac output syndrome and signs of pulmonary overcirculation. She was electively extubated without complications. An elevation in C-reactive protein was observed, although no pathogens were isolated in cultures. Follow-up echocardiography demonstrated patency of the anastomosis between the right pulmonary artery and the main pulmonary artery, as well as aortic reconstruction without stenotic gradients; there was no residual ductal shunt, left ventricular ejection fraction was 61%, and estimated pulmonary artery systolic pressure was 22 mmHg. Her ICU stay lasted 9 days, no additional procedures were required, and she was discharged 18 days after surgery (Table 1).

**Table 1:** Clinical variables

Variable	Case 01	Case 02
Preoperative details		
Age at symptom onset	5 days	15 days
Age at surgery	37 days	113 days
Weight	3.2 kg	3.4 kg
Sex	Female	Female
Place of origin	Chimbote (4 m a.s.l.)	Cajamarca (2750 m a.s.l.)
Symptoms	Interrupted feeding and cyanosis	Interrupted feeding and cyanosis
Physical signs	Grade III/VI systolic murmur at the lower left parasternal border with accentuated second heart sound	Grade II/VI systolic murmur at the left parasternal border, accentuated second heart sound
Diagnostic details		
AORPA type	Proximal AORPA	Proximal AORPA
Associated anomalies	PDA + ASD	PDA
Estimated pulmonary artery systolic pressure	60 mmHg	70 mmHg
Surgical details		
Procedure	End-to-side anastomosis of the RPA to the MPA + direct end-to-end repair of the ascending aorta	End-to-side anastomosis of the RPA to the MPA + direct end-to-end repair of the ascending aorta
Cardiopulmonary bypass time	134 min	120 min
Aortic cross-clamp time	76 min	65 min
Associated procedure	PDA ligation	PDA ligation
Intraoperative findings	RPA arising from the proximal posterior ascending aorta 12 mm above the valvular plane; PDA 8 mm; ASD 8 mm	RPA arising from the proximal ascending aorta 7 mm above the left coronary ostium; PDA 4 mm
Postoperative ICU stay		
Days on mechanical ventilation	8 days	10 days
Nitric oxide	No	No
Complications	Low cardiac output	Low cardiac output; bleeding; chylothorax
ICU length of stay	9 days	16 days
Immediate postoperative echocardiography		
Estimated pulmonary artery systolic pressure	22 mmHg	42 mmHg
Anastomotic stenosis	No	No
Left ventricular ejection fraction	61%	53%
Echocardiography at 16 months		
Estimated pulmonary artery systolic pressure	Not recorded	27 mmHg
Anastomotic stenosis	Not recorded	No
Left ventricular ejection fraction	Not recorded	77%

AORPA: anomalous origin of the right pulmonary artery from the ascending aorta, RPA: right pulmonary artery, PDA: patent ductus arteriosus, ASD: atrial septal defect, CPB: cardiopulmonary bypass, ICU: intensive care unit, MV: mechanical ventilation, ePASP: estimated pulmonary artery systolic pressure.

### Case 02

**Figure 4:**
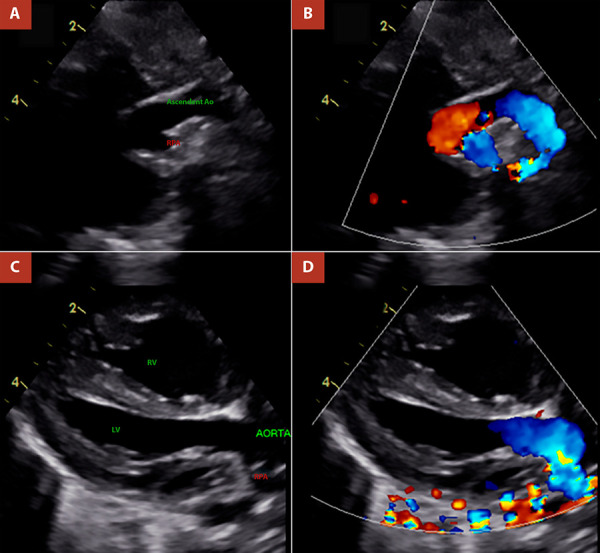
Transthoracic echocardiography, Case 02. **A)** and **B)** Suprasternal views showing the origin of the right pulmonary artery from the posterior wall of the ascending aorta. **C)** and **D)** Long-axis views demonstrating the origin of the right pulmonary artery from the ascending aorta. RPA: right pulmonary artery.

**Figure 5:**
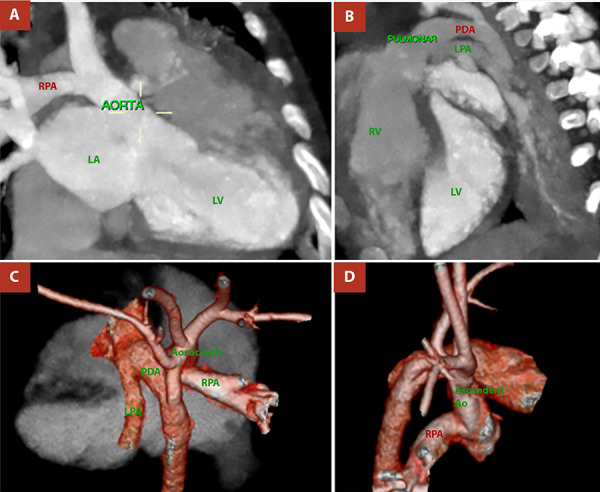
Cardiac and great-vessel computed tomographic angiography, Case 02. **A)** Anomalous origin of the right pulmonary artery (RPA) from the posterior wall of the ascending aorta. **B)** Origin of the left pulmonary artery (LPA) from the main pulmonary artery and presence of a patent ductus arteriosus (PDA). **C)** and **D)** Three-dimensional reconstruction of the heart and great vessels confirming the findings.

An infant was referred to our centre at 81 days of life with a presumptive diagnosis of TGA, and a history of respiratory distress, cyanosis, and interrupted breastfeeding since 15 days of life. On admission, vital signs included a heart rate of 124 beats per minute, respiratory rate of 48 breaths per minute, and oxygen saturation of 85%. Physical examination revealed retractions and a cardiac murmur on auscultation. Chest radiography demonstrated biventricular cardiomegaly with bilateral pulmonary overcirculation (Figure 1), while electrocardiography showed sinus rhythm with a QRS axis of +130°. Transthoracic echocardiography identified origin of the right pulmonary artery from the ascending aorta, a 4-mm patent ductus arteriosus, and an estimated pulmonary artery systolic pressure of 70 mmHg (Figure 4). Computed tomography findings were concordant with the echocardiographic results (Figure 5). The patient underwent surgical correction at 113 days of life, after completion of antibiotic therapy for sepsis.

**Figure 6:**
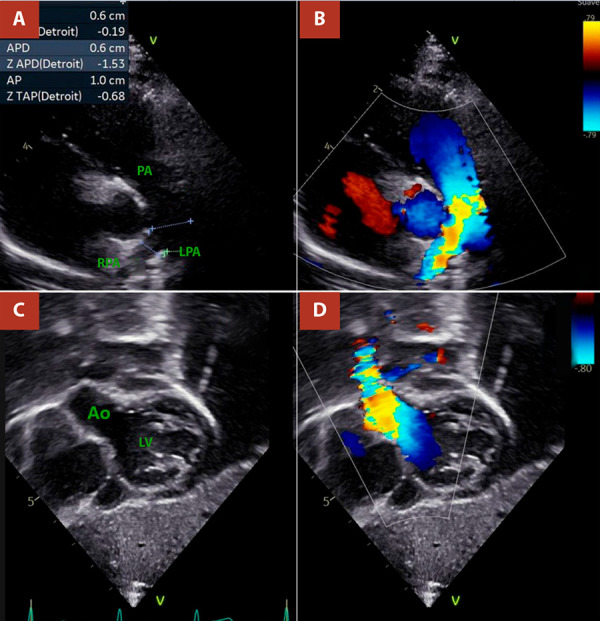
Follow-up echocardiography of Case 02 at 16 months. **A)** and **B)** Short-axis views showing no flow acceleration at the level of the pulmonary branches; right pulmonary artery diameter (z-score −1.5). **C)** and **D)** Subcostal view of the ascending aorta showing a reported maximum gradient <9 mmHg.

During her stay in the ICU, she developed low cardiac output syndrome, respiratory acidosis, and pulmonary overcirculation; additionally, she experienced haematuria, bleeding through the endotracheal tube, and chylothorax. She was electively extubated without complications. Follow-up echocardiography demonstrated patency of the anastomosis between the right pulmonary artery and the main pulmonary artery, as well as aortic reconstruction without stenotic gradients; a 3.5-mm atrial septal defect with left-to-right shunt was noted, with no residual ductal shunt, a left ventricular ejection fraction of 53%, and an estimated pulmonary artery systolic pressure of 42 mmHg. The ICU stay lasted 16 days, no additional procedures were required, and the patient was discharged 22 days after surgery. At 16 months of postoperative follow-up, serial echocardiographic evaluations showed no evidence of stenosis at the right pulmonary artery reimplantation site or at the supravalvular aortic level (Figure 6). Clinically, the patient has remained stable, with no signs of heart failure or cyanosis, although suboptimal weight gain has been reported, approximately −3.4 standard deviations for age (Table 1).

PA: pulmonary artery. RPA: right pulmonary artery. LPA: left pulmonary artery. LV. Left ventricle. Ao:aorta

### Surgical technique

Both cases were approached through a median sternotomy, with central aortobicaval cannulation, initiation of cardiopulmonary bypass (CPB), and induction of systemic hypothermia to 32°C. The patent ductus arteriosus was initially ligated, and following antegrade cardioplegia, the right pulmonary artery (RPA) was excised at its origin from the ascending aorta. Subsequently, the insertion site for the RPA was prepared on the right posterolateral aspect of the main pulmonary artery by means of a circumferential arteriotomy, after which a terminolateral (end-to-side) anastomosis between the RPA and the main pulmonary artery was performed. The ascending aorta was repaired using a direct terminoterminal (end-to-end) anastomosis (Table 1).

## Discussion

The anomalous origin of one of the pulmonary arteries is a rare condition that may involve either the right or left pulmonary artery. It has a low prevalence, with the most common subtype being AORPA, which comprises two forms: the proximal type (accounting for approximately 85% of cases), in which the right pulmonary artery originates close to the aortic valve, and the distal type, with origin near the innominate artery ^1^^,^^5^^,^^9^^,^^12^^,^^15^. This anomaly may be associated with other defects in up to 40% of cases ^3^, including atrial or ventricular septal defects, tetralogy of Fallot, interrupted aortic arch, aortopulmonary window, and most frequently patent ductus arteriosus (PDA) ^2^^,^^4^^,^^5^^,^^8^^-^^10^. The cases presented here correspond to the proximal right-sided type (proximal AORPA) associated with PDA.

From an embryological perspective, an abnormal truncal septation involving the fifth and sixth aortic arches has been proposed ^4^^,^^14^^,^^15^, although the exact mechanism remains uncertain. Because the aortic and pulmonary roots arise separately in this malformation, the term hemitruncus is considered a misnomer ^9^^,^^14^.

In this condition, the anomalous pulmonary artery functions as a large aortopulmonary collateral, exposing the vascular bed of the affected lung to systemic pressures and volume overload ^2^^,^^4^, while the pulmonary artery connected to the right ventricle receives the entire systemic venous return ^6^^,^^7^, thereby subjecting its pulmonary vascular bed to marked volume overload. These haemodynamic disturbances lead to the early development of heart failure and pulmonary hypertension ^2^, as observed in our patients, who also presented with cyanosis of multifactorial origin. In this specific pathology, cyanosis has been attributed in some reports to the marked elevation of pulmonary pressures ^3^^,^^12^^,^^16^. Electrocardiography showed right ventricular dominance, and chest radiography revealed cardiomegaly and pulmonary overcirculation, consistent with findings reported by other authors ^3^^,^^6^^,^^8^.

For diagnosis, echocardiography is usually sufficient, demonstrating the presence of two arterial roots, absence of the normal main pulmonary artery bifurcation pattern, and direct origin of the right pulmonary artery from the aorta ^1^^,^^16^. In the cases presented, the initial echocardiographic suspicion was TGA, due to the erroneous assumption that the vessel arising from the left ventricle was the bifurcating pulmonary trunk and that the vessel arising from the right ventricle was the aorta because it did not bifurcate, while overlooking the spatial relationship of the great vessels and coronary artery origins. In this context, correlation with cardiac computed tomography was particularly helpful ^5^. Similar initial echocardiographic misdiagnoses have been reported, such as in the series by Prifti *et al.*^10^.

In 1961, Armer et al. reported the first successful anatomic repair using a polyester fibre graft ^10^. Currently, several surgical techniques are available, and their application depends on intraoperative findings and surgeon preference ^1^^,^^9^^,^^11^. One such technique is direct end-to-side anastomosis between the right pulmonary artery and the main pulmonary artery, as performed in our patients, in whom a tension-free anastomosis was achieved ^12^^,^^17^. This required meticulous dissection of the right pulmonary artery, ascending aorta, and main pulmonary artery, as well as increased mobilisation of the latter by ligating and dividing ductal tissue. Regarding repair of the defect left in the ascending aorta after excision of the right pulmonary artery, the use of autologous or bovine pericardial patches has been suggested to prevent potential aortic stenosis ^17^. In the present cases, direct end-to-end repair was achieved without immediate evidence of right pulmonary artery compression or haemodynamic changes suggestive of coronary distortion, allowing uneventful separation from cardiopulmonary bypass with primary sternal closure and no need for early reintervention.

In the postoperative period, low cardiac output syndrome, pulmonary hypertension ^4^^,^^13^^,^^14^, and difficult extubation ^4^ are commonly expected. Our patients did not experience pulmonary hypertensive crises and were extubated without complications; however, both developed low cardiac output syndrome. In case 02, a coagulation disorder and chylothorax occurred, complications generally related to cardiac surgery rather than specifically to the underlying pathology. Follow-up echocardiography showed a reduction in estimated pulmonary artery systolic pressure.

In the long term, surgical or percutaneous reintervention may be required in 12.5% to 36% of patients to address supravalvular aortic stenosis or stenosis of the reimplanted pulmonary artery ^9^^,^^12^. Unfortunately, the first case involved a migrant patient who did not return for follow-up, and clinical evolution is unknown. The second case, with 16 months of clinical follow-up, remains clinically stable, albeit with poor weight gain. Follow-up echocardiography (Figure 6) showed no evidence of stenosis at the anastomotic sites; nevertheless, further evaluation with advanced imaging techniques, such as computed tomographic angiography or cardiac magnetic resonance, is warranted, as well as consideration of non-cardiac causes of inadequate weight gain.

In conclusion, AORPA is a rare entity whose diagnosis requires a high index of clinical suspicion and integration of detailed echocardiographic assessment with the support of cardiac and great-vessel computed tomographic angiography. Early surgical correction of this condition is feasible and can achieve favourable outcomes.
